# The complete genome and methylome of *Helicobacter pylori* hpNEAfrica strain HP14039

**DOI:** 10.1186/s13099-019-0284-y

**Published:** 2019-02-20

**Authors:** Binit Lamichhane, Eng-Guan Chua, Michael J. Wise, Connor Laming, Barry J. Marshall, Chin-Yen Tay

**Affiliations:** 1 Marshall Centre for Infectious Diseases Research and Training, School of Biomedical Sciences, University of Western Australia, Perth, WA Australia; 2 Department of Computer Science and Software Engineering, University of Western Australia, Perth, WA Australia; 3 Shenzhen Dapeng New District Kuichong People Hospital, Shenzhen City, 518119 Guangdong Province China

**Keywords:** Complete genome, Methylome, *Helicobacter pylori*, hpNEAfrica

## Abstract

**Background:**

*Helicobacter pylori* is a Gram-negative bacterium which mainly causes peptic ulcer disease in human, but is also the predominant cause of stomach cancer. It has been coevolving with human since 120,000 years and, according to Multi-locus sequence typing (MLST), *H. pylori* can be classified into seven major population types, namely, hpAfrica1, hpAfrica2, hpNEAfrica, hpEastAsia, hpAsia2, hpEurope and hpSahul. *Helicobacter pylori* harbours a large number of restriction-modification (R-M) systems. The methyltransferase (MTase) unit plays a significant role in gene regulation and also possibly modulates pathogenicity. The diversity in MTase can act as geomarkers to correlate strains with the phylogeographic origins. This paper describes the complete genome sequence and methylome of gastric pathogen *H. pylori* belonging to the population hpNEAfrica.

**Results:**

In this paper, we present the complete genome sequence and the methylome profile of *H. pylori* hpNEAfrica strain HP14039, isolated from a patient who was born in Somalia and likely to be infected locally during early childhood prior to migration. The genome of HP14039 consists of 1,678,260 bp with 1574 coding genes and 38.7% GC content. The sequence analysis showed that this strain lacks the cag pathogenicity island. The *vacA* gene is of S2M2 type. We have also identified 15 methylation motifs, including WCANHNNNNTG and CTANNNNNNNTAYG that were not previously described.

**Conclusions:**

We have described the complete genome of *H. pylori* strain HP14039. The information regarding phylo-geography, methylome and associated metadata would help scientific community to study more about hpNEAfrica population type.

**Electronic supplementary material:**

The online version of this article (10.1186/s13099-019-0284-y) contains supplementary material, which is available to authorized users.

## Background

*Helicobacter pylori* is a Gram-negative bacterium that colonises human stomach, resulting in various gastric diseases including gastritis, peptic ulcer disease and gastric cancer. About half of the world population is infected with *H. pylori* with prevalence in developing countries reaching up to 90% [1, 2]. *H. pylori* is well-known for its genome’s ability to mirror the history of human migration history due to its very long association with humans and transmission being within families [2–5]. This coevolution has led to the emergence of seven different MLST population types of *H. pylori,* based on the geographical origins, namely hpAfrica1, hpAfrica2, hpNEAfrica, hpEastAsia, hpAsia2, hpEurope and hpSahul [2, 3, 5].

*Helicobacter pylori* harbours large number of type II restriction-modification (R-M) systems [6–9]. The type II systems have separate DNA methyltransferase (MTase) and restriction endonuclease proteins (REase) that act on the same DNA sequence motif. Apart from protecting host DNA from foreign DNA, DNA methylation has been implicated in the regulation of bacterial gene expression [10]. The Type II RM systems in *H. pylori* are substantially diverse among strains and therefore can be used as a biomarker to trace geographical association of *H. pylori* [11, 12].

*Helicobacter pylori* strains of hpNEAfrica population type are mainly found circulating in Ethiopia, Somalia, Sudan and Nilo-Saharan speakers in northern Nigeria [2, 13]. No complete genome of a *H. pylori* strain originated from this region is available in public databases. Our complete genome and methylome data of *H. pylori* HP14039 hence will provide further indicators on the evolution and genetic diversity of this human gastric pathogen.

## Methods

### Bacterial culture and genomic DNA extraction

*Helicobacter pylori* strain HP14039 was isolated from a patient gastric biopsy sample onto selective agar plates. The selective plates used were Columbia blood agar plates (CBA) containing 5% horse blood (PathWest Laboratory Medicine WA Media, Australia) with Dent supplement (Oxoid, UK). The plates were incubated for 3–4 days at 37 °C in a 10% CO_2_ environment. The genomic DNA extraction was performed on 48 h bacterial culture using phenol–chloroform method [14]. Cells were harvested from culture plates and washed with PBS (pH 8) followed by centrifugation at 14,000 rpm for 1 min. Following the removal of supernatant, the pellet was resuspended in 50 µl of 0.5 M EDTA and 200 µl of sodium dodecyl sulphate and incubated at 50 °C for 2 h. Resultant lysate was thoroughly mixed with one volume 25:24:1 phenol:chloroform:isoamyl alcohol solution in a phase separating gel tube and spun at 14,000 rpm for 5 min; repeated once, then again subsequently with 24:1 chloroform:isoamyl alcohol. The aqueous layer was transferred to two volumes ice cold ethanol and gently mixed immediately. Precipitated DNA was then washed with 70% ethanol and solubilised in TE buffer. DNA quality and quantity were assessed using both Nanodrop (Thermofisher, USA) and Qubit (Thermofisher, USA).

### PacBio and Illumina MiSeq genome sequencing

The genomic DNA was sequenced using two sequencing platforms, Pacbio RSII and Illumina MiSeq. The PacBio sequencing was conducted by Novogene (HK) Co., Ltd, Hong Kong. For Illumina MiSeq sequencing, the genomic library was prepared using Nextera XT kit (Illumina, San Diego, USA) according to manufacturer’s protocol and sequenced using 2 × 300 paired-end protocol on an Illumina MiSeq instrument.

### Genome assembly and annotation

The Pacbio raw reads were assembled into a single contig using Canu assembler v1.7 [15], after which the assembly was circularized using Circlator v1.5.5 [16]. The circularised contig was subjected to further correction by mapping of Illumina MiSeq-generated paired-end reads using CLC Genomics Workbench 11 with default parameters (QIAGEN). Genome annotation was performed using Prokka v1.12 [17]. The annotation features are available in Additional file 1. Genome completeness and contamination of HP14039 genome was assessed using the taxonomy_wf workflow at species level available in CheckM v1.0.13.

### Processing of PacBio methylome data

All raw data in bax.h5 format were converted and merged into a bam file using bax2bam v0.0.8 prior to alignment to *H. pylori* HP14039 complete genome sequence using blasr v5.3.2 with default parameters. The aligned bam output file was then subjected to ipdSummary v2.3 to detect kinetic variations that were predictive of DNA modification events. Finally, the methylated DNA motifs were deduced using MotifMaker v0.3.1 [18, 19]. The density of methylated sites was plotted using Circos v0.69-6 with a window of 5000 bp [20].

### Phylogenetic analysis

The complete genome of HP14039, and 47 publicly available *H. pylori* complete genomes from NCBI database and 12 draft genomes of *H. pylori* strains isolated from our patients who were born in Northeast Africa, were used for core genome phylogeny analysis. The accession numbers of all *H. pylori* genomes used in this study are provided in Additional file 2: Table S1. For consistency, all genomes were annotated by Prokka v1.12 prior to using Roary v3.12.0 [21] for core genome analysis. In the Roary pipeline, sequence alignment of multiple core genes was performed using MAFFT v7.271 [22] and we specified that a gene must be present in all *H. pylori* strains to be considered as a core gene with the percentage identity cut-off of 95. The core alignment was then used to construct a neighbour joining tree using Mega v7.0.2 [23] and the output phylogenetic tree was visualized using Figtree v1.4.3 (http://tree.bio.ed.ac.uk/software/figtree).

### Quality assurance

Species confirmation was performed by using biochemical tests (urease, catalase and oxidase positives) and PCR with seven species-specific housekeeping genes (*atpA*, *efp*, *mutY*, *ppa*, *trpC*, *ureI* and *yphC*). Bacterial culture of pure single colony was used for genomic DNA extraction.

## Results and discussion

### Strain metadata and genomic characteristics

*Helicobacter pylori* strain HP14039 was isolated from the gastric biopsy of an Australian resident who was born in Somalia, located in the Northeast Africa region.

The genome of *H. pylori* HP14039 was sequenced using PacBio and Illumina technologies at 257× and 159× genome coverages, respectively. The final assembled genome is 1,678,260 bp in length with 1574 coding sequences, 36 tRNA genes and 38.7% G + C content. Genome assessment using CheckM revealed no contamination and 99.13% genome completeness. We found that it completely lacks the cag pathogenicity island, which is one of the major virulence factors and is thought to be associated with the development of gastric cancer [24]. Other major *H. pylori* virulence factors present in *H. pylori* HP14039 are listed in Table 1.

**Table 1:** Presence and absence of major virulence factors in HP14039 complete genome and other hpNEAfrica draft genomes (“+” means presence and “**–**” means absence)

Virulence factors	HP14039	HP01234	HP07036	HP08058	HP08061	HP08074	HP11049	HP13005	HP13050	HP13068	HP15005	HP98490	HP99255
*cag*-PAI	**–**	**–**	+	**–**	+	**–**	**–**	+^a^	**–**	+	**–**	+	**–**
*vacA*	+	+	+	+	+	+	+	+	+	+	+	+	+
*dupA*	+	+	**–**	**–**	**–**	+^b^	**–**	**–**	**–**	**–**	**–**	**–**	**–**
*iceA*	+	+	+	**–**	**–**	+	+	+	**–**	+	+	+	+
*babA*	+	+	+	+	**–**	+^b^	+	+^b^	+^b^	+^b^	**–**	+	**–**
*babB*	+	**–**	+	+	+^b^	+^b^	**–**	**–**	**–**	+^b^	+	**–**	**–**
*babC*	**–**	+	**–**	**–**	+^b^	**–**	**–**	**–**	**–**	**–**	**–**	**–**	+
*sabA*	+	+	+	+	+	+	+	+	+	+	+	+	+
*oipA*	+	+	+	+	+	+	+	+	+	+	+	+	+
*alpA*	+	+	+	+	+	+	+	+	+	+	+	+	+
*alpB*	+	+	+	+	+	+	+	+	+	+	+	+	+
*hopZ*	+	+	+	+	+	+	+	+	+	+	+	+	+
*napA*	+	+	+	+	+	+	+	+	+	+	+	+	+
*tieA/hp0986*	**–**	**–**	**–**	**–**	**–**	**–**	**–**	**–**	**–**	**–**	**–**	**–**	**–**
*ctkA/jhp0940*	**–**	**–**	**–**	**–**	**–**	**–**	**–**	**–**	**–**	**–**	**–**	**–**	**–**

^a^All *cag*-PAI elements are present, except *cagA*

^b^Truncated gene

### Methylome of HP14039

**Fig. 1:**
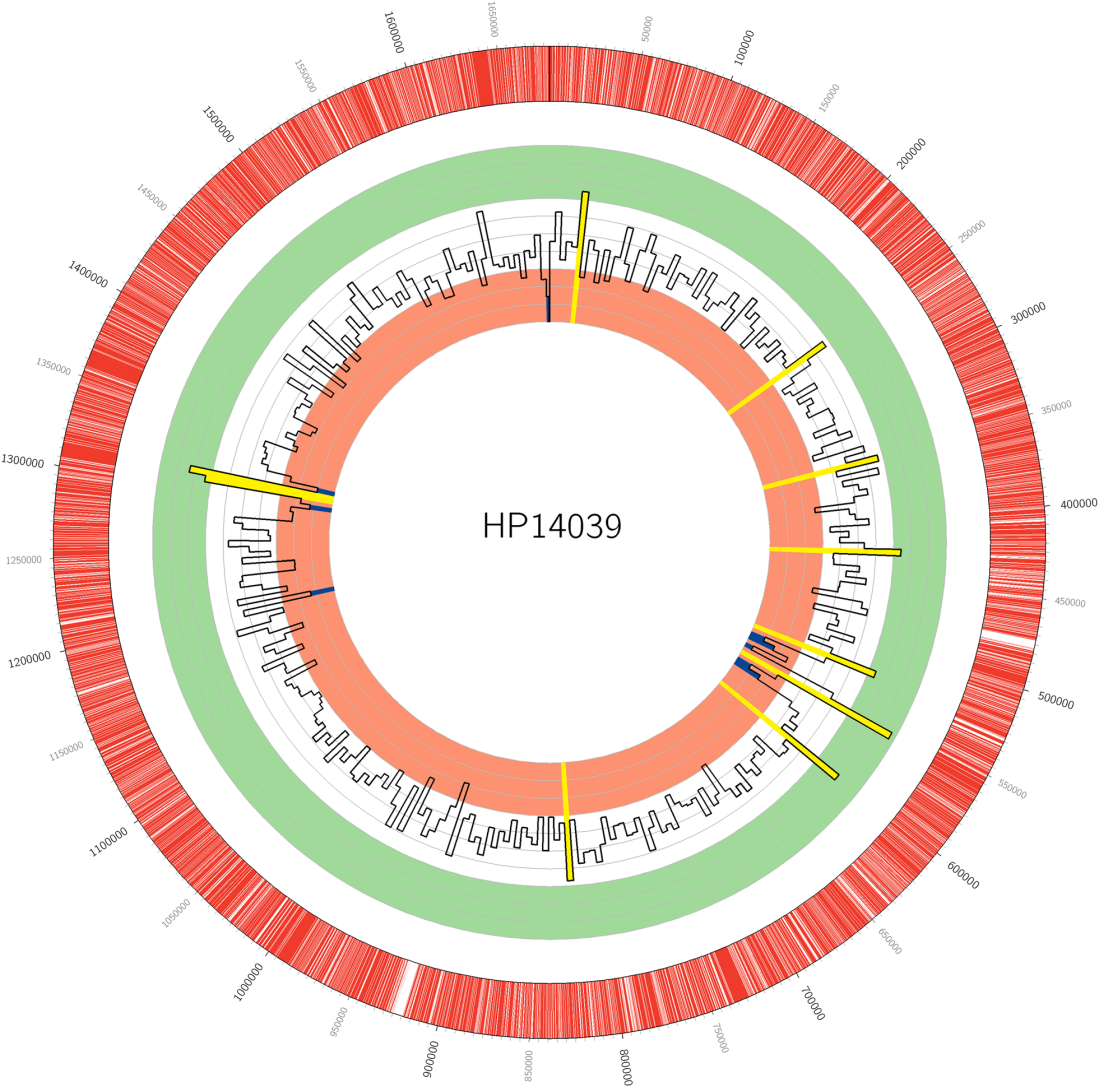
Circos plot displaying the density of methylated sites in HP14039 complete genome (5000 bp window). Open reading frames are highlighted in red in the outer ring. The inner histogram displays the abundance of methylated bases within every 5000 bp region over the chromosome, with an average of 367.9 ± 49.7. The hypermethylated and hypomethylated regions with methylated sites exceeding two standard deviations (> 466.7 and < 267.9, respectively) are highlighted in yellow and dark blue, respectively. The three outer green tracks within the histogram represent density values ranging from 600 to 481 (from outer to inner). The three inner orange tracks within the histogram represent density values ranging from 320 to 201 (from outer to inner)

Pacbio SMRT sequencing technology has the advantage of being able to detect the epigenetic state of sequenced DNA, and allow identification of modified nucleotides and methylated motifs. In HP14039 genome, a total of 62,407 methylated genomic positions were detected (m6A and m4C). The distribution of methylated bases over the HP14039 chromosome is presented in Fig. 1. Fifteen functional MTases were identified of which thirteen were assigned to their MTase genes based on previous studies [8, 25, 26]. Two methylated motifs, WCANHNNNNTG and CTANNNNNNNTAYG detected in this study were not described in earlier studies. All recognition sequence motifs and their corresponding MTases are summarised in Table 2.

**Table 2:** Methylated motifs detected in HP14039

Recognition site^a^	Modification type	No. detected	No. in genome	Detected (%)	Restriction component(s)			Methylation component(s)			Refs
					Locus tag	Ortholog	% identity	Locus tag	Ortholog	% identity	
5′-ACNGT	m4c	976	1112	87.8	HP14039_01407-01406	K747_11000	94.1, 93.9	HP14039_01408	K747_10995	95.5	[8]
5′-ATTAAT	m6a	949	952	99.7	HP14039_01410	jhp0431	94.1	HP14039_01411	jhp0430	94.9	[26, 29]
5′-CATG	m6a	14,872	14,874	100	HP14039_00716	hp1209	93.3	HP14039_00717	hp1208	93.8	[29]
5′-CCATC	m6a	2190	2199	99.6	HP14039_00375-00376	K747_12645	95.7, 92.7	HP14039_00372-00373	K747_03690	96.2, 97.7	[8]
5′-CCGG	m4c	3535	3542	99.8	HP14039_01604	hp0262	96.1	HP14039_01603	hp0263	94.2	[29]
5′-CTNAG	m4c	6164	6172	99.9	HP14039_01555	HpyHI	96	HP14039_01554	M.HpyHI	94.9	[30]
5′-GAGG	m6a	4672	4718	99	HP14039_00203^b^	–	–	HP14039_00204	hp0050	95.2	[26, 29]
5′-GATC	m6a	10,541	10,548	99.9	HP14039_00158	hp0091	93.7	HP14039_00157	hp0092	92.8	[29]
5′-GTAC	m6a	300	304	98.7	HP14039_01382-01381	jhp0455	93.4, 93.7	HP14039_01383	jhp0454	95.8	[26, 31]
5′-GTNNAC	m6a	820	826	99.3	HP14039_00987	hp0909	91.7	HP14039_00986	hp0910	96.1	[29]
5′-TCGA	m6a	610	612	99.7	–	–	–	HP14039_01606-01607	hp0260	96.1, 97.6	[32]
5′-TCNNGA	m6a	3795	3808	99.7	HP14039_00847-00846	jhp1013^c^	96.8, 97.5	HP14039_00848	jhp1012	96.1	[26]
5′-TGCA	m6a	11,060	11,072	99.9	HP14039_00305	HpyCH4 V	96.6	HP14039_00306	M.HpyCH4 V	93.4	[12, 30]
5′-CTANNNNNNNTAYG	m6a	191	192	99.5	–	–	–	HP14039_00325^d^	K747_03505	93	[8]
5′-WCANHNNNNTG	m6a	1732	4440	39	–	–	–	HP14039_01429^e^	K747_10905	92	[8]

^a^The methylated base within the motif is underlined while the modified base in the complementary strand is highlighted in red

^b^The 1–563 bp and 550–918 bp regions of HP14039_00203 demonstrated significant nucleotide sequence homology to the N-terminus of hp0052 and the C-terminus of hp0051, respectively

^c^Putative type II restriction enzyme probably recognising TCNNGA

^d^Predicted type I DNA methylase probably recognising CTANNNNNNNTAYG based on high sequence similarity with K747_03505 that recognises a closely related sequence motif, which is GANNNNNNNTAYG

^e^Predicted type I DNA methylase probably recognising WCANHNNNNTG. This gene is highly similar to K747_10905, which encodes a type I DNA methylase that recognises CCANNNNNNTC sequence motif

### Phylogeny

**Fig. 2:**
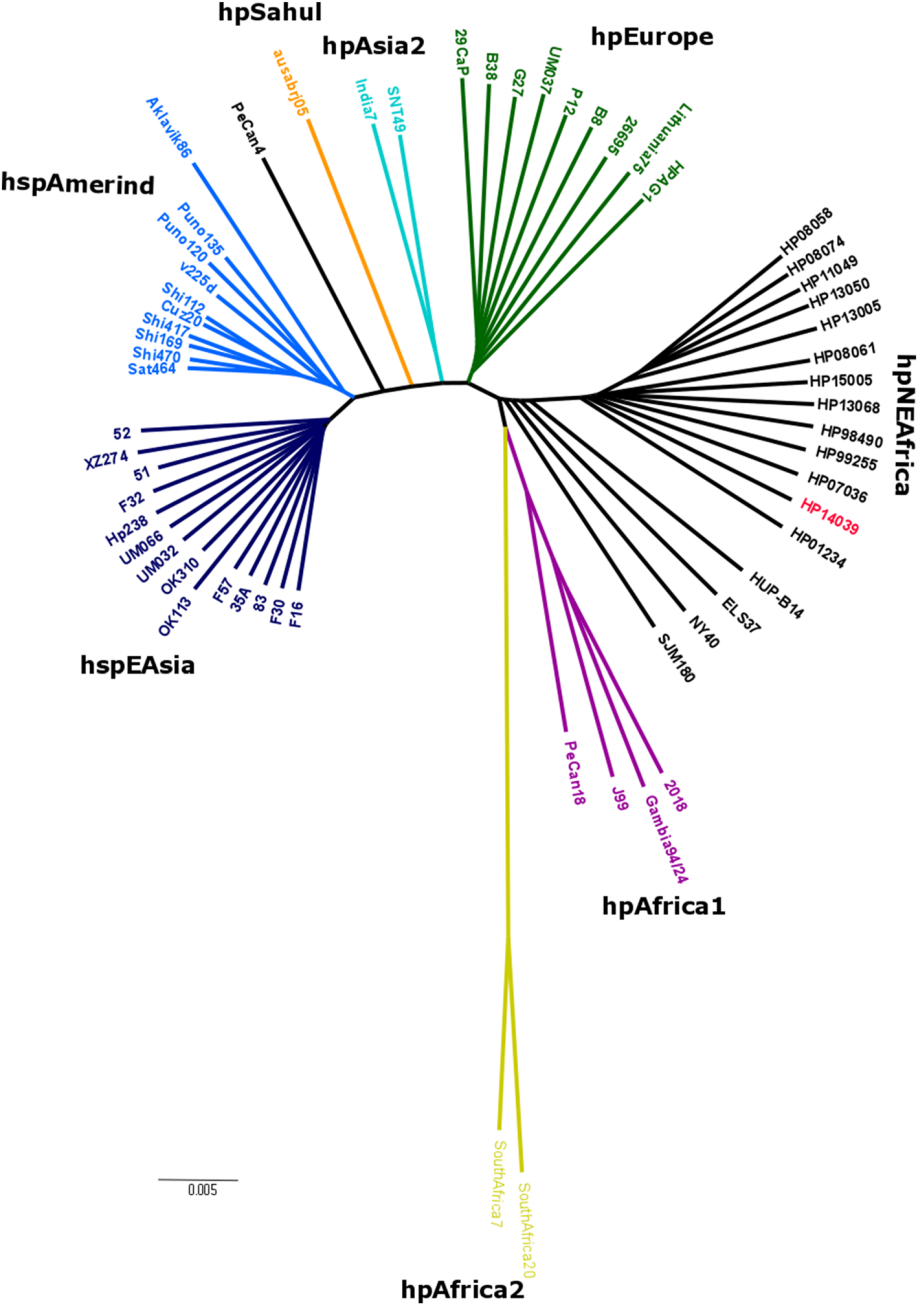
Core genome based phylogenetic tree of HP14039 with 47 complete genomes and 12 draft genomes. HP14039 is highlighted in red

The neighbour joining tree was constructed using core genome alignment derived from 48 complete *H. pylori* genomes including HP14039, and additionally 12 draft genomes of *H. pylori* strains isolated from patients originated from similar African region as HP14039. Among the 12 clinical strains that were included, two were from Somalia, identical to that of HP14039; four each from Sudan and Ethiopia, respectively; and the remaining two were from Eritrea. As *H. pylori* infection is common in early childhood [27], it is therefore highly likely that the patients have acquired these individual strains locally when young prior to their migration to Australia. The phylogenetic tree showed clear separation of *H. pylori* population types (Fig. 2). As expected, HP14039, along with other 12 clinical strains with similar geographical origins, were found clustered together. Importantly, HUP-B14, ELS37 and SJM180, which were isolated from Spain, El Salvador and Peru, respectively, were found to be closely related to hpNEAfrica and hpAfrica1 populations despite previous reports that these strains belong to the hpEurope population [28]. This indicates that the birthplace of the patient plays a more important and accurate role in determining the population type of a *H. pylori* isolate, than the geographical origin where the clinical isolate was acquired, as countless individuals are constantly migrating and moving in today’s globalised world.

## Additional files

**Additional file 1.** The original genbank file that shows annotation features of HP14039.

**Additional file 2: Table S1.** Strains used in this study to construct core genome phylogenetic tree.
